# Study on the isolation of rhizosphere bacteria and the mechanism of growth promotion in winter wheat in response to drought stress

**DOI:** 10.3389/fpls.2025.1595554

**Published:** 2025-08-19

**Authors:** Lijuan Zhang, Yanshuo Pan, Yanjie Qi, Jing Bai, Dongfei Han

**Affiliations:** ^1^ School of Environmental Science and Engineering, Suzhou University of Science and Technology, Suzhou, China; ^2^ Institute of Soil Science, Chinese Academy of Sciences, Nanjing, China; ^3^ School of Chemistry and Life Sciences, Suzhou University of Science and Technology, Suzhou, China

**Keywords:** wheat, PGPR, drought stress, inorganic phosphorus content, siderophore, comparative genomics

## Abstract

**Introduction:**

Wheat is one of the three major cereal crops in the world and is susceptible to the effects of drought stress. Rhizosphere microorganisms can affect plant growth by altering nutrient absorption and resistance to stress. Studying the plant–microbe interaction under drought stress to reveal the impact of soil microorganisms on plant growth in dry land has important scientific significance.

**Methods:**

In this study, seven plant growth-promoting bacteria were isolated from the rhizosphere soil of winter wheat, and their growth-promoting ability was compared and analyzed.

**Results:**

The results indicate that these strains are capable of hydrolyzing organic and inorganic phosphorus, fixing nitrogen, producing IAA (indole-3-acetic acid), ACC deaminase, and iron siderophore. Combined with pot experiment data, *Microbacterium* sp. I2, *Arthrobacter* sp. R4, and *Microbacterium* sp. K2 can significantly promote wheat growth. Under normal conditions, the wheat plant height increased by 5.17%, 13.02%, and 12.14% compared to the control group after one month of treatment with I2, R4, and K2, respectively. Under drought stress, the plant height increased by 6.41%, 2.56%, and -3.46%, respectively. However, under drought stress, only K2 significantly increased wheat root length by 11.94% compared to the control group. Therefore, K2 has stronger drought resistance than I2 and R4. Genome sequencing and comparative genome analysis of I2, R4, and K2 strains revealed that the strains contain functional gene clusters related to phosphorus solubilization (*pstABCS, phoUR*), ACC deamination (*accABD*), iron transport (*fepCDG*), IAA production (*trpABC*), nitrogen fixation (*nifUHJ*), drought resistance (*ostAB, treXYZ*), but with different gene types and copy numbers. Compared to I2, the R4 genome lacks one copy of the *phoUR* gene cluster, ACC deaminase, and iron transport related functional gene clusters. The K2 genome contains both *treXYZ* and *ostAB* gene clusters, which may be associated with its significant improvement in plant drought resistance.

**Discussion:**

This study indicates that PGPB may promote plant growth by affecting nutrient absorption and hormone synthesis, while also affecting plant drought resistance by regulating osmotic pressure and trehalose biosynthesis, providing a theoretical basis for regulation of plant growth in a sustainable way.

## Graphical Abstract

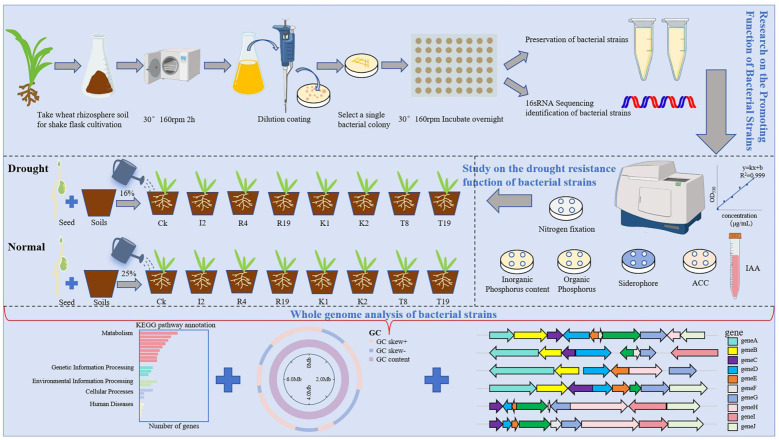

## 1 Introduction

Wheat is the second-largest cereal crop after rice, providing 20% of the calories consumed by the world’s population (Hassan et al., 2024). The yield of wheat has a direct and significant impact on the country’s food supply and food security (Sallam et al., 2019). However, during the growth and development process, wheat is susceptible to drought stress, leading to osmotic and oxidative stress, membrane degradation, and enzyme activity inhibition (Mohamed et al., 2024; Nunna et al., 2025), thereby altering the physiological and biochemical characteristics of plants and reducing crop yields.

In the past few years, plant growth-promoting rhizobacteria (PGPRs), which specifically refer to a specific bacterial community that colonizes the soil around plant roots or on the surface or inside roots, have received attention for their potential to increase crop yield (Bresson et al., 2013). Rhizosphere bacteria successfully established in soil ecosystems have high environmental adaptability and metabolic multifunctionality, promoting host growth through symbiosis with plants (Jochum et al., 2019). Due to the interaction between bacterial strains and hosts, PGPRs promote plant growth through various mechanisms, such as phosphorus solubilization, potassium solubilization, nitrogen fixation, quorum sensing (QS), siderophore production, 1-aminocyclopropane-1-carboxylate (ACC) deaminase production, volatile organic compound (VOC) production, plant hormone production, induction of systemic resistance, and promotion of beneficial plant–microbe symbiosis (Lee et al., 2024). PGPRs are of great significance in promoting plant growth and maintaining soil ecological health. Under drought stress, PGPRs can also alleviate damage to plants caused by drought by producing plant hormones or altering catalase activity. For example, previous studies have shown that PGPRs and arbuscular mycorrhizal fungi can enhance the activity of peroxidase in lettuce and improve its drought resistance (Naylor and Coleman-Derr, 2017). The ACC deaminase produced by PGPRs can degrade the precursor substance ACC of the endogenous plant hormone ethylene, enhancing the drought tolerance of plants such as tomatoes, peppers, and wheat (Grichko and Glick, 2001). Plant hormones produced by PGPRs, especially cytokinins, activate ABA-dependent signaling genes and enhance drought tolerance in sugarcane (Vargas et al., 2014). PGPRs play an important role in improving wheat drought resistance, which can enhance the antioxidant defense system of wheat under drought stress to protect wheat plants from oxidative stress. Previous studies have demonstrated that the inoculation of PGPRs in wheat plants under drought stress significantly enhances proline and lignin contents while strengthening the antioxidant defense system to protect the plants against oxidative stress. To mitigate the oxidative and osmotic stress caused by recurrent drought, PGPRs increase the relative water content (RWC) of inoculated plants by maintaining soil moisture within the rhizosphere. These growth-promoting strains are all high-quality producers of extracellular polysaccharides, which can form thick biofilms on the surface of roots, thereby helping to retain water even under drought conditions (Prasad et al., 2025). PGPRs enhance plant stress resistance under drought stress through a complex and interconnected collaborative network. They directly affect the physiological and biochemical processes of plants, improve their nutritional status and rhizosphere water conditions, and indirectly enhance the overall health and stress tolerance of plants by inducing systemic resistance and inhibiting pathogens.

Although the importance of studying rhizosphere microorganisms in wheat under drought conditions is evident, there are still some shortcomings at present. In particular, our understanding of the interaction between microorganisms and host plants under drought conditions is still not comprehensive. Therefore, in order to better cope with the impact of drought on wheat yield, it is urgent to deeply explore the rhizosphere microbial resources. The aim of this experiment was to investigate the effects of different PGPRs on wheat growth promotion indicators such as plant height and root length under drought stress. Combined with pot experiments, comparative genome analysis was conducted on three strains with good growth promotion and drought resistance abilities to explore the physiological mechanism of plant rhizosphere growth-promoting bacteria affecting wheat growth under drought stress and to provide a basis for the development and utilization of PGPRs in wheat drought resistance.

## 2 Materials and methods

Before starting this methodology section, we provide a summary flowchart (**Graphical Abstract**) to visually demonstrate the overall methodology used in this study.

### 2.1 Isolation of endophytic bacteria in winter wheat root

#### 2.1.1 Preparation of samples

The root tissue samples were washed with sterile water for 5 minutes, then 70% alcohol for 30 seconds, and a volume ratio of 1/100 sodium hypochlorite solution for 3 minutes. Finally, they were washed with sterile water five times, rinsed until clean, and cultured overnight on an Luria-Bertani (LB) solid culture medium. The absence of colony growth indicated that they have been thoroughly cleaned. Finally, the cleaned root samples were placed in a sterile water-only culture dish for overnight cultivation to restore endogenous bacteria.

#### 2.1.2 Sample grinding and isolation of strains

The clean root samples were ground, and 1 mL of suspension was taken and added to 99 mL of sterile water (or Phosphate Buffered Saline (PBS) buffer). They were cultivated at 30°C and shaken at 160 rpm for 2 hours. After inverted mixing, they were diluted to 10^−8^ in sequence. Three gradients of 10^−6^, 10^−7^, and 10^−8^ were selected, and 100 µL was applied to each of the three different Tryptic Soy Agar (TSA) plates. They were cultivated continuously for 2 weeks, and each colony was labeled (Beirinckx et al., 2020).

#### 2.2 Molecular biology identification of strains

The colonies were picked and cultured in 5 mL of liquid Trypticase (Tryptic) Soy Broth (TSB) medium at 25°C and 170 rpm for 48 hours. The genomic DNA of each culture was isolated using a bacterial DNA extraction kit (OMEGA Bacterial DNA Kit). The sequence of the 16S rRNA gene was amplified using the bacterial-specific primers, 27F (5′-AGAGTTTGATCCTGGCTCAG-3′) and 1492R (5′-GGTTACCTTGTTACGACTT-3′) (Asshauer et al., 2015). PCR amplifications were performed with 2 x Taq PCR Mix (Tiangen Biochemical Technology (Beijing) Co., Ltd.), 1.0 µM of each primer, genomic DNA as a template, and ddH_2_O for amplification. Three independent PCR amplifications were performed on each isolate for a total of 30 cycles, with 94°C denaturation (60 seconds), 55°C annealing (30 seconds), 72°C elongation (60 seconds), and finally 72°C elongation (10 minutes). The resulting PCR products were sequenced using a platform (sinogene ax, China, Sangon Biotech (Shanghai)Co. Ltd.). The taxonomy of each isolate was confirmed by the alignment of these amplified sequences with the NR database in GenBank with BLAST and the comparison with nucleotide sequence homology of the 16S rRNA gene for bacteria (Wannawong et al., 2024). A phylogenetic tree based on 16S rRNA gene sequences was constructed using the neighbor-joining method.

### 2.3 Plant growth-promoting traits of bacterial isolates

#### 2.3.1 Nitrogen fixation

The nitrogen fixation potential of purified bacterial strains was evaluated through cultivation in a nitrogen-depleted growth medium. The isolated bacteria were inoculated in a nitrogen-free medium (5.0 g L^−1^ mannitol or glucose, 0.2 g L^−1^ KH_2_PO_4_, 0.2 g L^−1^ MgSO_4_·7H_2_O, 0.2 g L^−1^ NaCl, 0.2 g L^−1^ CaSO_4_·2H_2_O, 5 g L^−1^ CaCO_3_, 15 g L^−1^ agar, and 1 L of distilled water, pH = 7.0–7.2) (Fu et al., 2022). These cultures were incubated on the nitrogen-free medium at 30°C for 5 days. The formation of a bacterial colony was defined as nitrogen fixation ability. The amplification of the *nifH* gene was performed on strains with nitrogen fixation ability, and the reaction process was as follows: 95°C for 1 minute, 58°C for 1 minute, and 72°C for 1 minute, for a total of 35 cycles. PCR products were detected through electrophoresis using 1% agarose gel.

#### 2.3.2 Phosphate solubilization

Soil suspensions of 10^−6^, 10^−7^, and 10^−8^ were continuously diluted, and 100 μL was transferred to phosphate agar plates and cultivated at 28°C for 4 days (Surange et al., 1997). Single colonies with obvious halos were selected for the quantitative measurement of phosphates. To quantify the soluble phosphate produced by the strains, a 500-μL bacterial culture was inoculated into 50 mL of inorganic phosphorus culture medium and incubated at 28°C and 180 rpm for 4 days, and the soluble P concentration was determined in the culture supernatant using the colorimetric molybdate blue method. One milliliter of supernatant was placed into a colorimetric tube and diluted to 50 mL, 1 mL of 10% ascorbic acid and 2 mL of molybdate chromogenic reagent were added to the reaction system, and a 30-second vortex oscillation was performed to ensure thorough mixing. Immediately after the dark reaction termination stage, they were incubated at room temperature in the dark for 15 minutes, and finally, a UV–visible spectrophotometer was used for quantitative detection at the characteristic wavelength of 700 nm.

#### 2.3.3 Siderophore production

According to the method of Schwyn and Neilands (1987), the blue agar chrome azurol S (CAS) medium was used to characterize the siderophore production potential of all bacterial isolates (Alexander and Zuberer, 1991). The quantification of the siderophore produced from each isolated strain was performed in a liquid medium supplemented with CAS. The isolates with a yellowish-orange halo were further inoculated into a liquid Modified King B (MKB) medium and incubated at 30°C and 180 rpm for 3 days. Then, cell-free supernatants from the culture were collected by centrifugation (10,000 × *g* for 5 minutes, 30°C), and 3 mL of the bacterial supernatant was added to the same amount of CAS solution, followed by dark incubation for 1 hour. Sterile water was used as a blank control for comparison and calculation. All treatments were performed in triplicate. The absorbance of the isolates was measured at 630 nm using a spectrophotometer. The ratio As/Ar (OD_630_ of treatments/OD_630_ of blank control) represented the capacity of producing siderophores.

#### 2.3.4 Indole-3-acetic acid production

The bacterial strain culture, maintained overnight at 28°C and 120 rpm in a TSB medium, was inoculated into 50 mL King B medium containing 100 mg L^−1^ l-tryptophan at a ratio of 1:100 (v/v). The bacterial culture was incubated at 28°C and 120 rpm for 7 days and then centrifuged at 1,000 rpm for 10 minutes to obtain the supernatant. The indole-3-acetic acid (IAA) concentration was determined using the Salkowski colorimetric technique. An equal amount of bacterial supernatant was taken and reacted with the Salkowski chromogenic reagent (1:1, v/v) in the dark for 30 minutes (28°C). The unvaccinated culture medium was used as a blank control, and spectrophotometric detection was performed at a wavelength of 530 nm, with three replicates in each group (Glickmann and Dessaux, 1995).

#### 2.3.5 1-Aminocyclopropane-1-carboxylate deaminase activity assay

The bacterial cultures were incubated on the Dworkin-Foster Minimal Medium (DF) solid medium at 28°C for 7 days. The ability of the strains to produce ACC deaminase was indicated by the formation of bacterial colonies. Subsequently, if growth was observed, the strains were transferred to the liquid medium containing ACC as the sole nitrogen source and cultured for one additional day. An ACC concentration gradient standard curve was established in a 96-well plate, and the ACC content was quantitatively analyzed in the 96-well plate boiling water bath system using the high-throughput ninhydrin colorimetric method (Li et al., 2011).

### 2.4 Pot experiments

#### 2.4.1 Wheat seed germination

Seed pretreatment: Seeds with plump and evenly sized particles were selected, disinfected in a NaClO solution with a volume ratio of 2.5/100 for 2 minutes, shaken, disinfected in 75% ethanol for 60 seconds, rinsed two times with sterile distilled water for 30 seconds each time, and air-dried at room temperature for 5 minutes. Seed germination was carried out using the plate culture method (Fu et al., 2025). A volume of 15 mL of different bacterial suspensions (the supernatant was centrifuged, discarded, and mixed with sterile water) was inoculated into wheat seeds (ZXM 99) and incubated at 28°C for 3 days in the dark on a plate covered with double-layer filter paper. Seeds treated with equal volumes of distilled water under the same conditions were used as the control (Combs-Giroir et al., 2024).

#### 2.4.2 Drought stress treatment

The soil was filtered through a 2-mm nylon sieve and disinfected at 121°C for 20 minutes in a high-pressure sterilization pot. Seedlings treated using the above method were planted in 800 g of sterilized soil for each pot. According to the field experiment conducted in Hebei Province in the early stage, the drought was 60% of the field capacity with a moisture content of approximately 16%, while the control was 80% of the field capacity with a moisture content of approximately 25%. Each treatment was replicated three times. All seedlings were grown in a greenhouse at 25°C (16 hours of light and 8 hours of darkness). Four weeks later, growth indicators such as plant height and root length of seedlings were observed and recorded under different treatments. After washing the wheat roots, the shape was stretched, and the height of the aboveground part and the length of the main root were accurately measured using a ruler method.

### 2.5 Genome sequencing and annotation

#### 2.5.1 Library construction and genome sequencing

DNA samples were extracted from bacterial strains using the MP Genomic DNA Kit for testing. Briefly, DNA samples (initial DNA quantity, 200 ng) were sheared into ~400-bp fragments using a 50W Covaris M220 Focused Acoustic Shearer following the manufacturer’s protocol and ultrasound for 65 seconds. Illumina sequencing libraries were prepared from the sheared fragments using the NEXTFLEX Rapid DNA-Seq Kit. The 5′ primer ends were first end-repaired and phosphorylated. Next, the 3′ ends were A-tailed and ligated to sequencing adapters. The third step is to enrich the adapter-ligated products using PCR. The prepared libraries were then used for paired-end Illumina sequencing (2 × 150 bp) on Illumina NovaSeq 6000 (or other new sequencers) (Illumina Inc., San Diego, CA, USA). The specific steps are as follows: using chemically modified DNA polymerase and four fluorescently labeled dNTPs, only a single base was incorporated in each cycle. By scanning the surface of the reaction plate with a laser, the types of nucleotides bound to each template chain in the first round of reaction were detected. Subsequently, the fluorescent label and termination group were removed through a chemical reaction to restore the activity of the 3′ hydroxyl group, allowing for the next round of nucleotide extension. By collecting and analyzing fluorescence signals one by one, the base sequence of the template DNA was ultimately determined.

#### 2.5.2 Genome assembly and annotation

All analyses were performed using the online platform of Majorbio Cloud Platform (www.majorbio.com) from Shanghai Majorbio Bio-Pharm Technology Co., Ltd (Ren et al., 2022). The detailed procedures were according to the method of Yoon et al. (2017). Raw reads obtained after sequencing were filtered using the fastp software (version 0.19.6), followed by assembly with SOPA *de novo* version 2.04. The paired-end Illumina reads were trimmed of adaptors, and low-quality reads (length <50 bp or with a quality value <20) were removed using fastp (Chen et al., 2018) (https://github.com/OpenGene/fastp, version 0.23.0). The final assembled genome was submitted to the NCBI database (Dahar et al., 2024). Glimmer (Delcher et al., 2007) was used for Coding sequence (CDS) prediction, tRNA-scan-SE was used for tRNA prediction, and Barrnap (https://github.com/tseemann/barrnap) was used for rRNA prediction. The predicted CDSs were annotated from the NR, Swiss-Prot, Pfam, GO, COG, KEGG, and CAZy databases using sequence alignment tools such as BLASTP, Diamond, and HMMER (Kanehisa et al., 2022). Briefly, each set of query proteins was aligned with the databases, and the annotations of best-matched subjects (e-value < 10^−5^) were obtained for gene annotation. Based on the comprehensive score value of the comparison, the one with the highest value was selected as the optimal matching result. Biosynthetic gene clusters (BGCs) of secondary metabolites were identified using the antiSMASH v5.1.2 software. Other parameters are default on the antiSMASH official website (https://antismash.secondarymetabolites.org/#!/start). Artificially screened genes promote plant growth- and drought-related functions. The sequences of drought-resistant and growth-promoting genes were downloaded from the NCBI database, and protein homology alignment with existing genes was performed.

### 2.6 Drought tolerance determination of bacterial strains

Using PEG6000 (polyvinyl alcohol) to simulate a dry environment, I2, R4, and K2 seed solutions were cultivated in a turbid state at 28°C and 170 rpm for 2 days and centrifuged at 1,000 rpm for 5 minutes, and the supernatant was discarded. After mixing the bacterial cells with sterile water three times, 0.5% of the bacterial culture was inoculated into the TSB liquid medium containing PEG6000 with mass concentrations of 5, 10, 15, 20, and 30 g/100 mL. Three parallel samples were taken for each concentration of the liquid culture medium, and the optical density (OD) value at 600 nm was measured after 48 hours of cultivation at 37°C and 170 rpm.

### 2.7 Statistical analysis

Statistical analysis was based on the SPSS 26.0 software. Single-factor analysis of variance was used between multiple groups, and p < 0.05 was considered statistically significant. Significantly different averages are represented by different letters.

## 3 Results

### 3.1 Strains isolated from wheat rhizosphere soil

**Figure 1:**
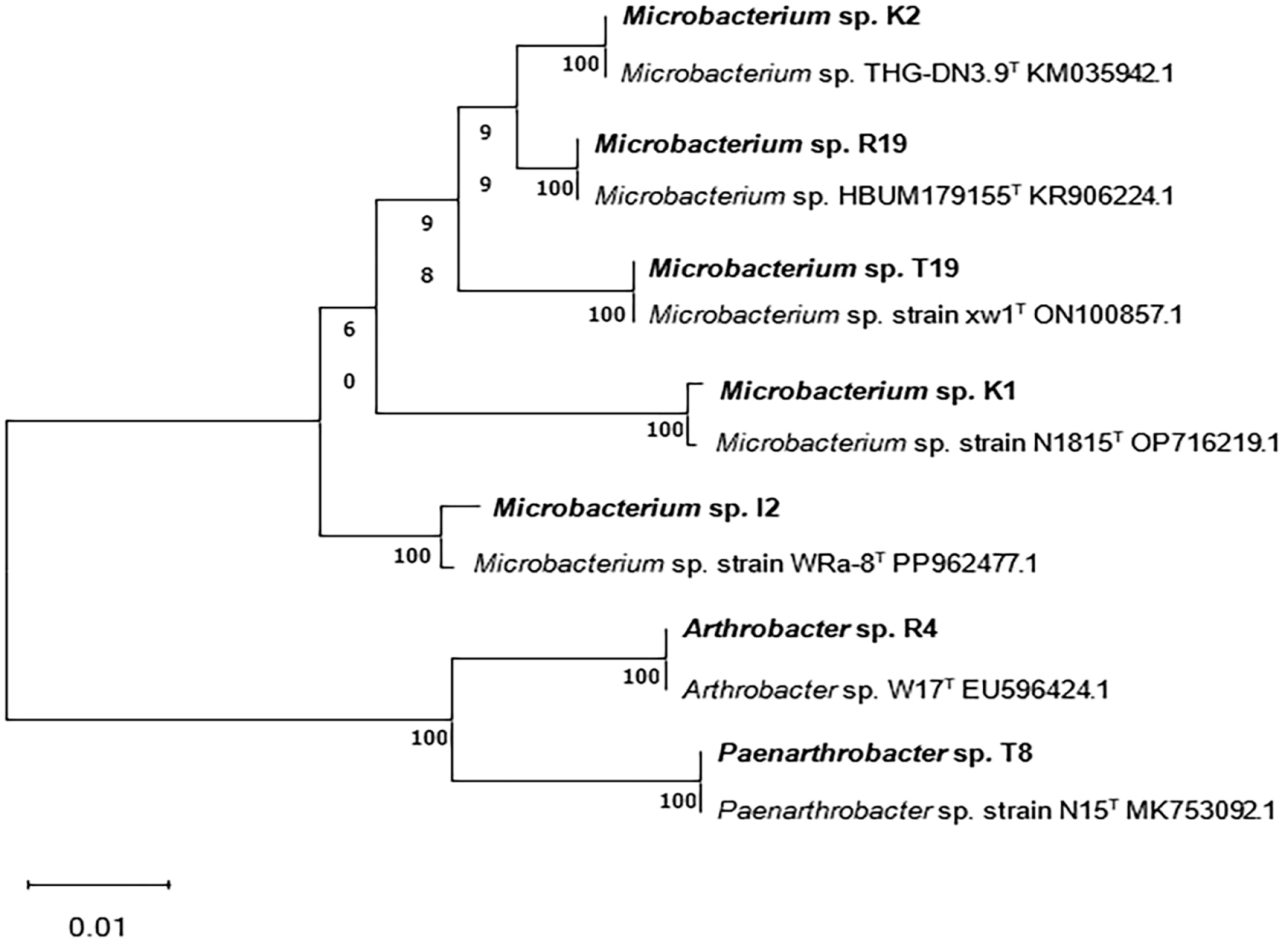
Phylogenetic tree based on 16S rRNA gene sequences of isolated strains *Microbacterium* sp. I2, *Arthrobacter* sp. R4, *Microbacterium* sp. R19, *Microbacterium* sp. K1, *Microbacterium* sp. K2, *Paenarthrobacter* sp. T8, and *Microbacterium* sp. T19.

According to the full-length sequence of the 16S rRNA gene, seven isolated bacterial strains were identified using 16S rRNA gene homology >97% as the standard. Based on the closest species in the sequences of the 16S rRNA gene, the seven isolates were named *Microbacterium* sp. I2, *Arthrobacter* sp. R4, *Microbacterium* sp. R19, *Microbacterium* sp. K1, *Microbacterium* sp. K2, *Paenarthrobacter* sp. T8, and *Microbacterium* sp. T19. The results showed that the PCR electrophoresis band positions of the isolated strains were consistent with those of the experimental strains, and all strains belonged to the phylum Actinobacteria (**Supplementary Figure S1**). The phylogenetic relationship based on the full sequences of 16S rRNA genes of all seven bacterial strains confirmed their taxonomy (**Figure 1**).

### 3.2 The potential of the isolated strain to promote plant growth

The plant growth-promoting potential of seven isolated bacterial strains was characterized *in vitro* by determining qualitatively and/or quantitatively the traits phosphate solubilization, nitrogen fixation, ACC deaminase, IAA, and siderophore production. It was found that strains I2 and R4 had relatively strong growth-promoting ability (**Table 1**).

**Table 1:** Plant promotion potential of seven strains of bacteria.

Strain	Nitrogen fixation	Organic phosphorus	Inorganic phosphorus content (mg L^−1^)	Siderophore production (%)	ACC deaminase activity	IAA production (µg mL^−1^)
*Microbacterium* sp. I2	+	+	31.83 ± 1.59b	37.32a	+	2.88 ± 0.14bc
*Arthrobacter* sp. R4	+	+	19.83 ± 0.99c	26.06a	+	1.34 ± 0.07c
*Microbacterium* sp. R19	+	–	–	–	–	3.94 ± 0.20ab
*Microbacterium* sp. K1	+	–	–	6.34b	–	5.27 ± 0.26a
*Microbacterium* sp. K2	+	+	17.62 ± 0.88c	4.93b	+	2.64 ± 0.13bc
*Paenarthrobacter* sp. T8	+	+	–	–	+	5.13 ± 0.26a
*Microbacterium* sp. T19	+	–	51.81 ± 2.59a	–	+	1.48 ± 0.07c

Different lowercase letters in the same column indicate significant differences (p < 0.05).

+, capable; −, incapable; ACC, 1-aminocyclopropane-1-carboxylate; IAA, indole-3-acetic acid.

#### 3.2.1 Nitrogen fixation

The nitrogen fixation ability of strains I2, R4, R19, K1, K2, T8, and T19 was judged through their apparent growth on the nitrogen-free medium (**Supplementary Figure S2**). The amplification results of the nitrogenase *nifH* gene of the strains are shown in **Supplementary Figure S3**, using *Streptomyces lincolnensis* L4 containing the *nifH* gene as a positive control and *Escherichia coli* as a negative control (Fu et al., 2022). According to the results, a PCR product matching the expected size of *nifH* was amplified from strains I2, R4, and K2, which is consistent with the genome sequencing results. The *nifH* gene sizes of I2, R4, and K2 were 1,433 (gene1278), 1,682 (gene1192), and 1,118 bp (gene1170), respectively. Among them, the PCR product that matched the size of *nifH* amplified from strain R4 was clearer, while the PCR products from I2 and K2 were weaker. Except for T8, none of the other strains showed any *nifH* amplification of approximately 1,500 bp, which may be due to a primer sequence mismatch due to variations in *nifH* genes among different species.

#### 3.2.2 Phosphate solubilization

The phosphorus solubilization ability of the strains was determined based on whether they generate a phosphorus solubilization circle (**Supplementary Figure S4**). There were four strains—I2, R4, K2, and T19—with the ability to hydrolyze inorganic phosphorus, and T19 had the strongest ability to hydrolyze inorganic phosphorus, reaching 51.81 mg L^−1^.

#### 3.2.3 Siderophore activity

Strains I2, R4, K1, and K2 can form an orange halo on the CAS medium, and quantitative detection was performed. SU represents siderophore activity, with SU = [(Ar − As)/Ar] * 100. The larger the SU, the stronger the strain’s ability to produce siderophore (**Supplementary Figure S5**). Due to the lack of a single siderophore (or compound) that can serve as a standard for various microbial siderophores for standard curve determination, the quantitative detection used in this method was only a relative quantification that reflects the ability of different bacterial strains to produce siderophores. The siderophore activity of strains I2 and R4 was relatively stronger than that of K1 and K2, with 37.32% and 26.06%, respectively (**Table 1**).

#### 3.2.4 Indole-3-acetic acid production

All strains had the ability to produce IAA, and the concentration of IAA produced by strain K1 was the highest, reaching 5.27 ± 0.26 µg mL^−1^. Strain R4 had the weakest ability to produce IAA, with only 1.34 ± 0.07 µg mL^−1^. The IAA synthesis ability of strains I2 and K2 was relatively close, with IAA concentrations of 2.88 ± 0.14 and 2.64 ± 0.13 µg mL^−1^, respectively (**Table 1**).

#### 3.2.5 1-Aminocyclopropane-1-carboxylate deaminase activity

Strains I2, R4, K2, T8, and T19 were grown well on the DF medium supplemented with ACC as the sole nitrogen source, indicating their ACC deaminase activity for decomposing ACC into ACC butanoic acid and ammonia, which could be utilized as direct nitrogen sources for plants (**Supplementary Figure S6**). The quantitative measurement revealed significant differences among the five positive strains in their ability to produce ACC deaminase, with ACC concentration lower than that in the control group. Notably, strain R4 demonstrated the lowest ACC concentration, indicating superior ACC deaminase activity compared to the other strains (**Supplementary Figure S7**).

### 3.3 Effect of strains on wheat growth under soil culture conditions

#### 3.3.1 Effect of strains on plant height and root length in wheat

**Figure 2:**
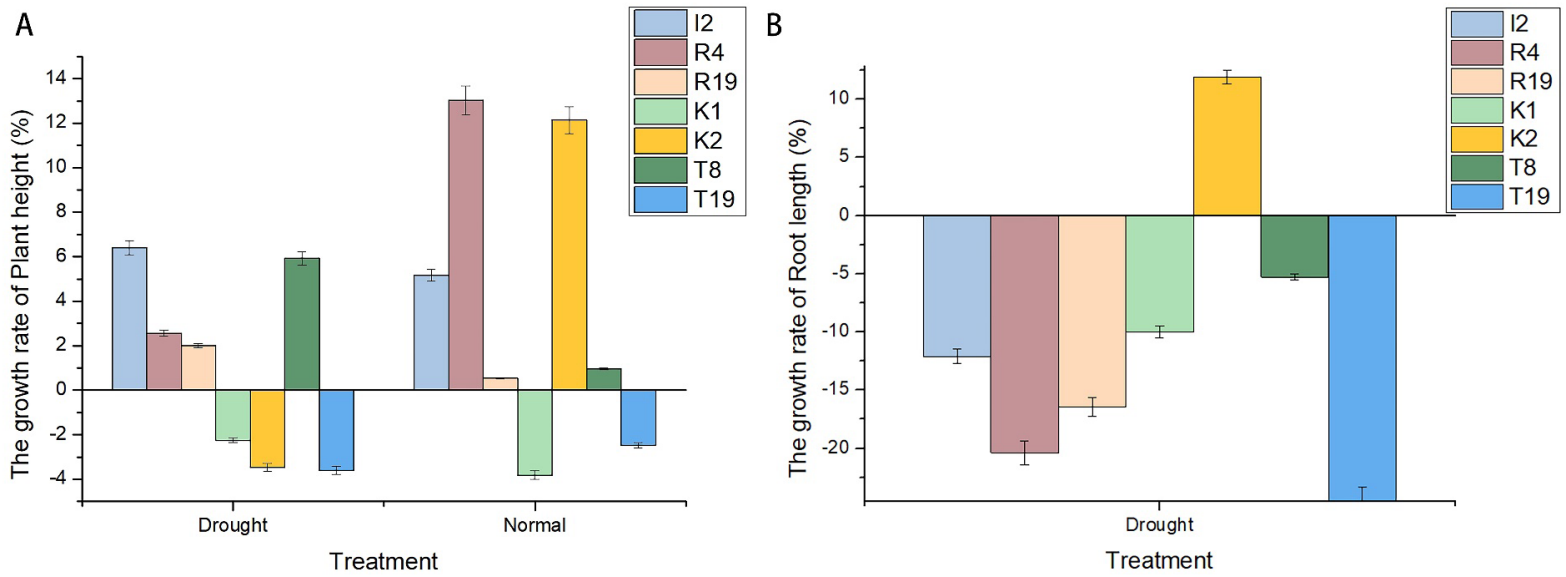
Changes in both plant and root height and length growth rates of *Microbacterium* sp. I2, *Arthrobacter* sp. R4, *Microbacterium* sp. R19, *Microbacterium* sp. K1, *Microbacterium* sp. K2, *Paenarthrobacter* sp. T8, and *Microbacterium* sp. T19. **(A)** shows varying positive plant height growth rates with higher values under normal conditions. **(B)** shows negative root length growth rates under drought, with a few positive values under normal conditions.

Under normal conditions, the wheat plant height increased by 5.17%, 13.02%, and 12.14% compared to the control group after 1 month of treatment with I2, R4, and K2, respectively; under drought stress, the plant height increased by 6.41%, 2.56%, and −3.46%, respectively. However, under drought stress, only K2 significantly increased wheat root length by 11.94% compared to the control group (**Figure 2**). Therefore, K2 has great application potential to improve wheat drought resistance.

### 3.4 Genome analysis of bacterial strains

From the assembled draft genome of strain I2, the estimated genome length (3,957,576 bp), protein-encoding DNA sequence (3,894 CDSs), plasmid count (0 plasmid), and predicted rRNA (including 47 tRNAs, 2 rRNAs, and 9 sRNAs) were identified. From the assembled draft genome of strain R4, the estimated genome length (4,299,747 bp), protein-encoding DNA sequence (4,467 CDSs), plasmid count (five plasmids), and predicted rRNA (including 51 tRNAs, 5 rRNAs, and 23 sRNAs) were identified. From the assembled draft genome of strain K2, the estimated genome length (3,436,560 bp), protein-encoding DNA sequence (3,540 CDSs), plasmid count (0 plasmid), and predicted rRNA (including 44 tRNAs, 4 rRNAs, and 9 sRNAs) were identified (**Supplementary Table S1**).

**Figure 3:**
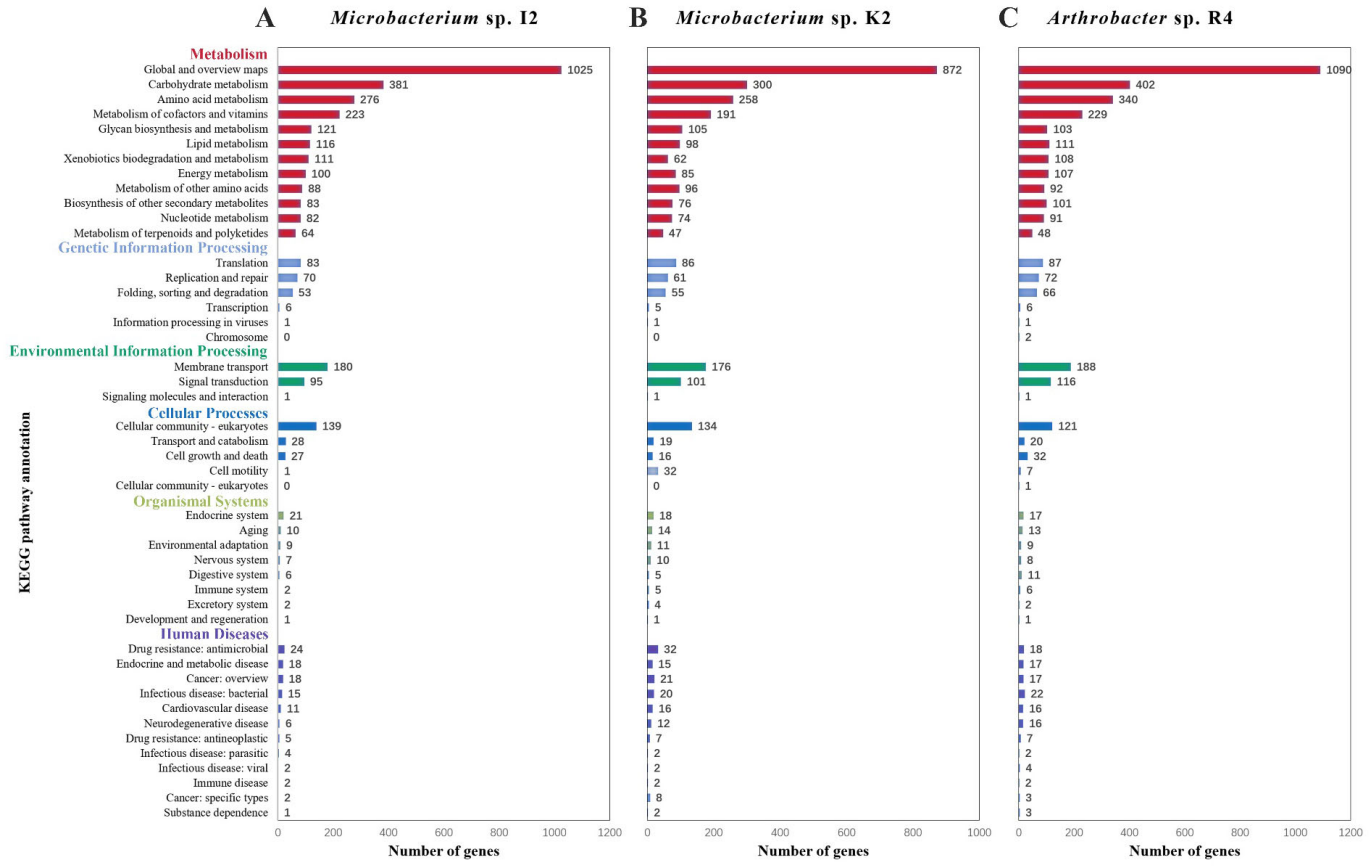
KEGG pathway annotation of different strains. **(A)** Genomic annotation maps of strains Microbacterium sp. I2 based on KEGG. **(B)** Genomic annotation maps of strains *Microbacterium* sp. K2 based on KEGG. **(C)** Genomic annotation maps of strains *Arthrobacter* sp. R4 based on KEGG.

From the function annotation of KEGG of I2, in addition to two major categories of central carbon metabolism of “carbohydrate metabolism” (381 CDSs) and “amino acid metabolism” (276 CDSs), other characteristic categories included “xenobiotics biodegradation and metabolism” (111 CDSs), “metabolism of terpenoids and polyketides” (64 CDSs), and “biosynthesis of other secondary metabolites” (83 CDSs) (**Figure 3A**). In addition to the central carbon metabolism “carbohydrate metabolism” (402 CDSs) and “amino acid metabolism” (340 CDSs), the characteristic categories of R4 included “xenobiotics biodegradation and metabolism” (108 CDSs), “metabolism of terpenoids and polyketides” (48 CDSs), and “biosynthesis of other secondary metabolites” (105 CDSs) (**Figure 3B**). In addition to the central carbon metabolism of “carbohydrate metabolism” (300 CDSs) and “amino acid metabolism” (258 CDSs), these characteristic categories of K2 included “xenobiotics biodegradation and metabolism” (62 CDSs), “metabolism of terpenoids and polyketides” (47 CDSs), and “biosynthesis of other secondary metabolites” (76 CDSs) (**Figure 3C**).

**Figure 4:**
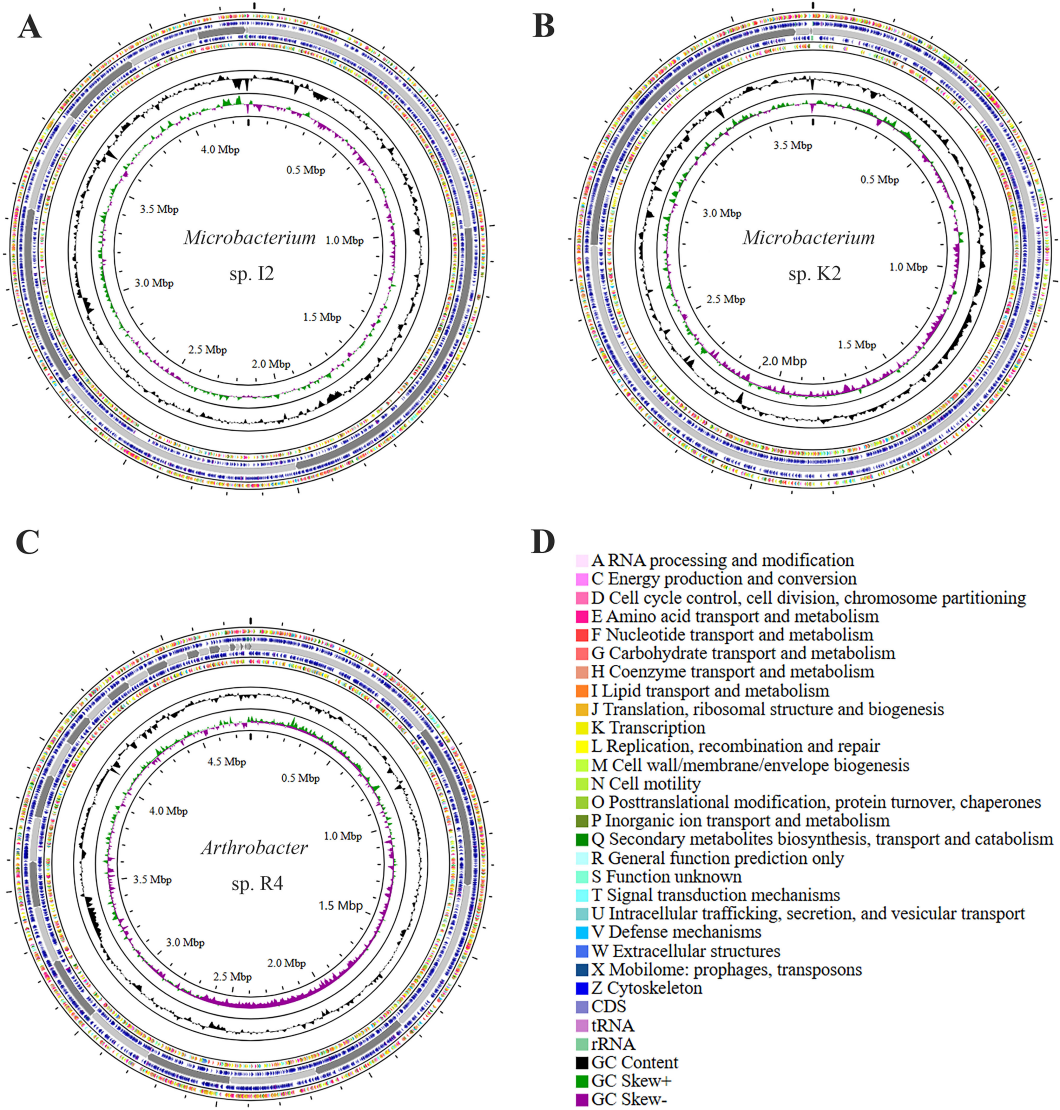
Genomic map of strains *Microbacterium* sp. I2, *Arthrobacter* sp. R4, and *Microbacterium* sp. K2: the outermost circle is the indication of the genome size. The second and third circles are genes on the positive and negative strands of the genome, respectively. Different colors represent different COG functional classifications. The fourth circle is the repeat sequence. The fifth circle is tRNA and rRNA. The sixth circle is the GC content. The innermost circle is GC skew. A–Z show the functional classification of the CDS genes in the chromosome.

Circos visualizes the genome, which can be used to more clearly explore the relationship between genome components or locations (**Figure 4**). The different colors in **Figure 4** represent the different functions of genes annotated in the COG database, including amino acid transport metabolism, general function prediction, secondary metabolism biosynthesis, transport, and catabolism. The genome sequences of strains I2, R4, and K2 have all been submitted to NCBI GenBank, and the accession numbers are JBLWIH000000000, JBLWIJ000000000, and JBLWII000000000, respectively.

### 3.5 Analysis of genes related to plant growth promotion and drought resistance in bacterial strains

Through whole genome sequencing, we have identified a series of genes that may be involved in growth promotion, including nitrogen fixation, phosphorus fusion, ACC deamination, iron transport, and IAA synthesis.

#### 3.5.1 Phosphate solubilization

**Figure 5:**
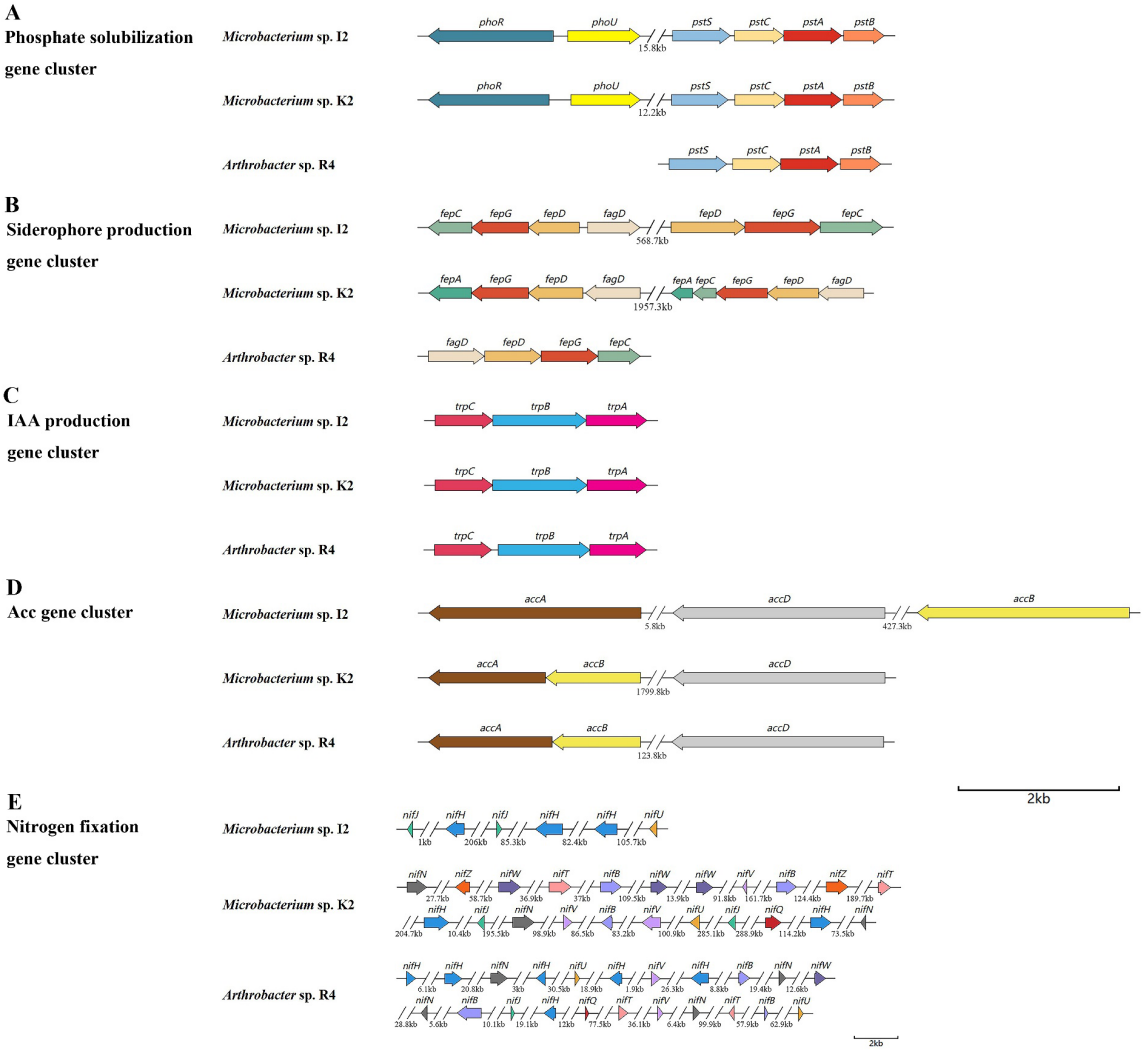
Gene clusters related to promoting plant growth.

Phosphorus is essential for the synthesis of nucleic acids, coenzymes, and ATP in plants. However, most of the phosphorus in the soil cannot be absorbed by plants. Many rhizosphere bacteria are capable of converting these phosphates into usable forms (Xie et al., 2016). We identified a total of 16 key genes related to phosphate solubilization in the genomes of strains I2, K2, and R4 (**Supplementary Table S2**), which can assist in the transport of various substrate molecules in the phospholipid bilayer by overcoming concentration gradients. Phosphate transporters encoded by *pstS*, *pstC*, *pstA*, and *pstB* were found in the phosphate metabolism pathway (**Figure 5**). The *pstB* gene maintained a homology level of 80%–90% among the three strains. The *pstC* gene exhibited highly conserved characteristics (>80%). In contrast, only I2 and K2 of the *pstS* (phosphate-binding protein PstS) and *pstA* (phosphate transport system permease protein PstA) genes had sequence homology exceeding 60% (**Table 2**).

**Table 2:** Homologous genes involved in promoting plant growth and drought resistance in the genomes of strains *Microbacterium* sp. I2, *Arthrobacter* sp. R4, and *Microbacterium* sp. K2.

Function	Gene name	I2 Gene ID	I2 and K2 identity (%)	K2 Gene ID	I2 and R4 identity (%)	R4 Gene ID	K2 and R4 identity (%)
Nitrogenase iron protein	*nifH*	gene1278	NA	gene1170	NA	gene1192	NA
Permease PstA	*pstA*	gene0409	64.05	gene0221, gene1531, gene3268	55.86	gene1017	52.34
ATP-binding protein PstB	*pstB*	gene0410	89.58	gene3581, gene3267	83.40	gene1018	80.70
Permease subunit PstC	*pstC*	gene0408	67.52	gene3269	62.13	gene1016	61.98
Phosphate ABC transporter substrate-binding protein PstS	*pstS*	gene0407	60.54	gene3270	47.43	gene1015	47.85
Iron-enterobactin ABC transporter ATP-binding protein	*fepC*	gene2421, gene3149	58.82, 54.55	gene1920, gene3582	64.31, 54.79	gene4449	61.22
Fe^3+−^ siderophore ABC transporter permease	*fepD*	gene2419, gene3151	70.67, 44.90	gene1922, gene3584	52.65, 45.27	gene2914, gene4447	51.33, 49.33
Iron-enterobactin ABC transporter permease	*fepG*	gene2420, gene3150	71.31, 39.72	gene1921, gene3583	43.62, 38.46	gene4448	45.56, 36.53
Probable siderophore-binding lipoprotein YfiY	*fagD*	gene3152	32.23, 34.56	gene1923, gene3585	27.91	gene4446	43.38, 29.67
Tryptophan synthase alpha chain	*trpA*	gene2329	74.81	gene0542	53.38	gene2990	56.25
Tryptophan synthase beta chain	*trpB*	gene2328	87.94	gene0541	68.99	gene2989	67.23
Indole-3-glycerol phosphate synthase	*trpC*	gene2327	77.73	gene0540	55.65	gene2988	51.42
Trehalose-6-phosphate synthase	*ostA*	gene3210, gene1956	74.78	gene0753	63.07	gene0009, gene0173	61.29
Trehalose-phosphate phosphatase A	*ostB*	gene1957	49.01	gene0752	40.84	gene0008, gene4332	42.40

#### 3.5.2 IAA

Nine key genes responsible for acetic acid production (*trpA*, *trpB*, *trpC*, *trpD*, *trpS*, and *trpE*) and a series of related genes (*iaaB*, *iaaF*, and *iaaL*) were identified in the genomes of three strains (**Supplementary Table S2**). Among them, genes *trpA*, *trpB*, and *trpC* were able to synthesize gene clusters (**Figure 5**), emphasizing the ability of the bacteria to produce IAA. The gene sequence homology between I2 and K2 was 74.81% in *trpA*, 87.94% in *trpB*, and 77.73% in *trpC* (**Table 2**). The homology was higher in R4 and K2 (56.25%, 67.23%, and 51.42%) and R4 and I2 (53.38%, 68.99%, and 55.65%), which was consistent with the data of IAA concentration measured in I2 and K2, which were higher in R4 (**Table 1**).

#### 3.5.3 Nitrogen fixation

Genes related to nitrogen fixation were also identified in the strain. Unfortunately, we did not discover a complete nitrogenase gene cluster. Three genes related to MoFe protein (*nifH*, *nifJ*, and *nifU*) were identified in the genomes of three strains; *nifW*, *nifN*, *nifV*, *nifQ*, and *nifZ* were deficient in the genome of I2 (**Supplementary Table S2**). The annotation indicates that these genes are associated with nitrogenase activity and are responsible for biological nitrogen fixation.

#### 3.5.4 ACC deaminase

The genomes of R4 and K2 were found to contain an ACC deaminase synthetic gene cluster, which is composed of two genes (*accA* and *accB*) (**Figure 5**). Compared to I2, they lack a set of copies of the ACC deaminase synthesis gene cluster.

#### 3.5.5 Siderophore

The genes related to encoding siderophore in the genome sequence of bacterial strains were classified. Genes related to enterobacterin transporters (*fepA*, *fepB*, *fepC*, *fepD*, *fepG*, and *fagD*) were discovered (**Supplementary Table S2**). By comparing the common genes (*fepC*, *fepD*, *fepG*, and *fagD*) among the three strains, we found that the homology of the *fepC* gene between I2 and R4 and the homology of the *fepG* gene between I2 and K2 were relatively high at 64.31% and 71.31%, respectively, while the homology between other genes was below 60% (**Table 2**). The siderophore enterobactin is recognized by the outer membrane receptor *fepA*. Once enterobactin passes through the outer membrane, the periplasmic binding protein (PBP) *fepB* will transport it to the inner membrane. On the inner membrane, the complex composed of *fepC*, *fepD*, and *fepG* will transport enterobactin to the cytoplasm (Dong et al., 2019). *sirA*, which is responsible for siderophore transport, was classified in the genomes of three strains. SirA is located outside the cell membrane, which can capture iron-bound siderophores and help bacteria acquire iron.

#### 3.5.6 Drought tolerance

The key genes related to drought resistance in strains were classified, and the homologous similarity was compared. The biosynthetic pathways of trehalose vary in different organisms. In the drought stress environment, the content of trehalose in plants will significantly increase, improving their drought resistance. At present, there are five known biosynthetic pathways, namely, the OtsA–OtsB pathway, TreP pathway, TreS pathway, TreY/TreZ pathway, and TreT pathway.

**Figure 6:**
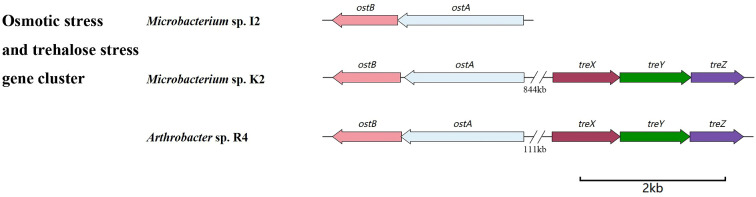
Gene clusters related to drought resistance.

The *ostA* (6-phosphate trehalose synthase, OstA) and *ostB* (trehalose phosphatase, OstB) gene clusters were identified in the genomes of three strains (**Figure 6**). The sequence homology of *ostA* among the three strains was higher than that of *ostB* (**Table 2**). The genes related to sugar ABC transporter substrate binding genes (*thuE* and *thuF*) were also classified (**Supplementary Table S2**), which are membrane channel proteins that utilize the energy generated by ATP hydrolysis in the cytoplasm to regulate the efflux/influx of substances. They can affect both membrane protein function and the energy system within the cell membrane and, therefore, can be modified to regulate membrane permeability. This strategy has the potential to simultaneously improve strain growth, increase substrate utilization, and increase strain productivity.

**Figure 7:**
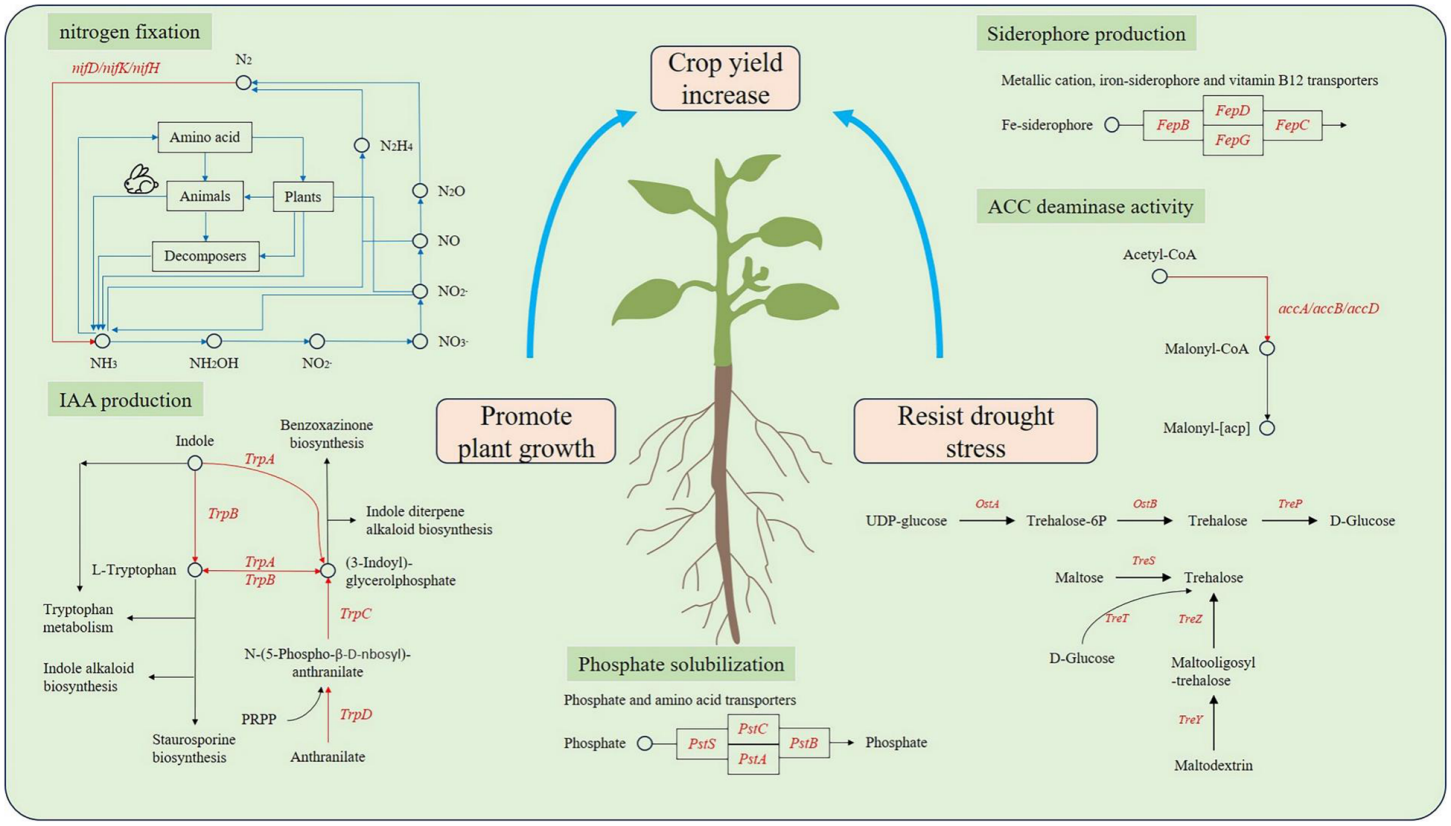
Speculative model diagram of plant growth promotion and drought resistance mechanisms.

In addition, *treX*, *treY*, and *treZ* are clustered in the R4 and K2 genomes but are missing in the I2 genome (**Figure 6**). There are TreP and TreT trehalose synthesis pathways in the R4 and K2 genomes (**Supplementary Table S2**). In the TreY/TreZ pathway, maltose trehalose synthase (*treY*) catalyzes the conversion of maltodextrin to maltose trehalose, followed by maltose trehalose hydrolase (*treZ*), which hydrolyzes maltose trehalose to form trehalose (**Figure 7**).

### 3.6 Drought tolerance determination of bacterial strains

Drought tolerance analysis was conducted based on the size of OD_600_ of the strain in a TSB liquid medium with different concentrations of PEG6000. The results are shown in **Supplementary Figure S6**. All three strains grew well in a PEG6000 medium with a mass concentration of 5–30 g/100 mL, indicating that the strains have a certain degree of drought tolerance. As shown in **Supplementary Figure S8A**, under simulated drought conditions with PEG6000 at a mass concentration of 10%–15%, the OD_600_ value of the strain did not significantly decrease but showed no significant difference from the 0% OD_600_ value. This indicates that strain I2 has good adaptability to mild drought. As shown in **Supplementary Figure S8B**, with the increase of PEG6000 mass concentration, the OD_600_ value of strain R4 also decreases, which is consistent with the growth situation of most bacteria, indicating a decrease in activity with the increase of environmental drought. As shown in **Supplementary Figure S8C**, under the condition of 5%–10% PEG6000, the absorbance value of strain K2 is significantly higher than that of 0% PEG6000, indicating that under simulated drought conditions of 5%–10%, there is no significant effect on the growth of strain K2, but it is beneficial for its survival and growth.

## 4 Discussion

The promotion of plant growth may result from the beneficial functions of applying PGPR strains, such as producing plant growth hormones, nitrogen fixation, and phosphorus solubilization (Hassen et al., 2018). Moreover, it was previously demonstrated that PGPRs could continue to contribute to improved plant growth even when subjected to both drought and heat stress simultaneously due to the cumulative effect of these mechanisms (Bashan and Levanony, 1990; Notununu et al., 2022). It was also highlighted that a diverse microbial population provides multiple pathways for improving plant health and productivity (Ates and Kivanc, 2020). These microorganisms with the ability to promote plant growth and development will be the best alternative to fertilizers (Hassen et al., 2018). This study focuses on the screening and isolation of PGPRs and their effects on the growth of winter wheat. The molecular biology identification indicated that the seven isolates in total belonged to the phylum Actinobacteria. Multiple studies have proven the function of Actinobacteria in plant growth promotion (Jog et al., 2012; Ning et al., 2019).

ACC deaminase can produce a certain amount of α-ketone butyrate ester, which has been proven in other PGPR strains (Penrose and Glick, 2003; Checcucci et al., 2017; Jaemsaeng et al., 2018). The genomes of strains I2, K2, and R4 contained related gene clusters. In addition, the genes involved in tryptophan metabolism in the KEGG metabolic pathway have also been identified, which is closely related to the synthesis of amino acetoacetic acid (Asaf et al., 2018). The *iaaB*, *iaaF*, and *iaaL* genes were all present in three strains (I2, K2, and R4), while the *trpABCDES* genes were only present in strain R4. One of the abundant sources of phosphate in soil is in the form of phosphonates, and the phosphonate gene cluster is responsible for the bacterial degradation of phosphonates, which releases biologically available phosphate for nearby plants (Asaf et al., 2018). In addition, the Phosphate Specific Transport (PST) system is a high-affinity, low-speed, free Pi transport system, and some phosphate metabolism genes such as *phoU*, *phoA*, *pstA*, *pstB*, *pstC*, and *pstS* are believed to have phosphate solubilization ability (Iqbal et al., 2021; Wang et al., 2022). Through the function annotation of three bacterial strains, we found that these genes (encoding phosphate transporters) collectively constitute the phosphate transport system. In terms of nitrogen fixation, we only found nitrogen fixation-related genes *nifU*, *nifQ*, *nifH*, *nifB*, etc., in individuals. The *nifU* protein mainly plays a role in Fe–S cluster aggregation. It is necessary for nitrogen fixation. Siderophore is a relatively low-molecular-weight (500–1,000) organic chelating agent that combines with insoluble iron in the environment to form Fe^3+−^ siderophore complexes. Bacteria that produce siderophores change the utilization rate of iron in soil through the chelation of siderophores, thereby increasing the utilization rate of iron in the plant rhizosphere and meeting the nutritional needs of plants for iron (Garzon-Posse et al., 2019). In this study, we identified a siderophore synthesis gene cluster (*fepC*, *fepD*, *fepG*, and *fagD*) in the genomes of three bacterial strains. The results of the activity test of siderophore showed that the strains of I2, R4, and K2 had the ability, with 37.32%, 26.06%, and 4.93%, respectively.

In the above research results, the plant growth-promoting assay suggested that all seven isolates have the potential to promote growth, of which the I2, R4, and K2 strains have better promotion effects. All three strains showed positive results across six performance indicators. Among them, I2 had the highest inorganic phosphorus content and a siderophore activity, with 31.83 mg L^−1^ (37.32%). In addition, under non-drought stress in the pot experiments, the plant height of winter wheat treated with I2 and K2 seed dressing was significantly higher than that of the control group, which increased by 5.17% and 12.14%, respectively. Further, in the analysis of related functional gene clusters, it was found that the similarity of functional genes between I2 and K2 was higher than that in R4, and the type and copy number of functional genes related to phosphorus dissolution, iron carrier activity, and ACC deaminase activity were higher than those in R4. This further explains the molecular mechanism in which I2 and K2 have stronger growth-promoting ability than R4.

Drought stress seriously affected the yield of wheat, and the content of trehalose in the plant was closely related to its drought resistance (Yeo et al., 2000; Hwang et al., 2004). Drought stress experiments showed that the plant height of wheat treated with drought stress was significantly lower than that of the control group (data not shown). After adding strains, the negative effect on plant height was alleviated (**Figure 2**). Under normal conditions, the wheat plant height increased by 5.17%, 13.02%, and 12.14% compared to that of the control group after 1 month of treatment with I2, R4, and K2, respectively; under drought stress, the plant height increased by 6.41%, 2.56%, and −3.46%, respectively. However, under drought stress, only strain K2 significantly increased root length by 11.94% compared with that in the control. The longer taproot of wheat can penetrate into the soil and absorb water from deeper soil layers, thus helping wheat maintain water balance and enhance its drought resistance. Functional gene mining showed that trehalose biosynthesis pathway-related genes existed in the three strains, but the biosynthesis pathways were different. Only the OstA–OstB pathway was present in I2, but four different trehalose synthesis pathway genes were in K2; this may explain the fact that I2 and K2 exhibited different effects on root length and plant height in wheat under drought stress. Moreover, there may be more unknown genes associated with drought stress to be identified.

## 5 Conclusion

We have confirmed from the aspects of growth-promoting function verification, plant phenotype determination, genome analysis, etc., that the isolated strains have the traits of nitrogen fixation, phosphorus solubilization, siderophore activity, ACC deaminase activity, and IAA production. The results emphasized the potential of natural PGPRs as a biological inoculant to improve wheat productivity and water stress resilience (Salem et al., 2024). Therefore, it is necessary to explore the methods and mechanisms of the stable positive effects of plant rhizosphere growth-promoting bacteria in practical applications so that more PGPRs can be used in the future to solve the related problems encountered by wheat growth and development in dry land. This will provide a more sustainable and environmentally friendly approach in the agricultural field, reduce the demand for chemical pesticides and fertilizers (Timmusk et al., 2014), and help to improve crop yields and farmers’ economic benefits.

## Data Availability

The data presented in the study are deposited in the NCBI repository, accession numbers are respectively JBLWIH000000000 JBLWII000000000 and JBLWIJ000000000.
