# Supplementary material for: Study on the isolation of rhizosphere bacteria and the mechanism of growth promotion in winter wheat in response to drought stress
Source: Front Plant Sci. 2025 Aug 19;16:1595554. doi: 10.3389/fpls.2025.1595554 (PMC12406708; doi:10.3389/fpls.2025.1595554)
Supplement: Supplementary file 1 [file DataSheet1.docx]

# **Supplementary Figure legends**

Supplementary Figure 1. Electrophoretic map of amplified DNA fragments from bacterial strains using primer 27F/1492R.

Note: M in the figure: DL2000 DNA Marker; 1: *Microbacterium* sp. I2; 2: *Arthrobacter* sp. R4; 3: *Microbacterium* sp. R19; 4: *Microbacterium* sp. K1; 5: *Microbacterium* sp. K2; 6: *Paenarthrobacter* sp. T8; 7: *Microbacterium* sp. T19.

Supplementary Figure 2. Determination of nitrogen fixation ability of strains *Microbacterium* sp. I2, *Arthrobacter* sp. R4, *Microbacterium* sp. R19, *Microbacterium* sp. K1, *Microbacterium* sp. K2, *Paenarthrobacter* sp. T8 and *Microbacterium* sp. T19.

Supplementary Figure 3. PCR amplification of nitrogenase *nifH* gene of strain.

Note: M in the figure: DL2000 DNA Marker; 1: positive control; 2: negative control; 3: *Microbacterium* sp. I2; 4: *Arthrobacter* sp. R4; 5: *Microbacterium* sp. R19; 6: *Microbacterium* sp. K1; 7: *Microbacterium* sp. K2; 8: *Paenarthrobacter* sp. T8;

9: *Microbacterium* sp. T19.

Supplementary Figure 4. Determination of Organophosphorus Capacity of Strains *Microbacterium* sp. I2, *Arthrobacter* sp. R4, *Microbacterium* sp. K2, *Paenarthrobacter* sp. T8.

Supplementary Figure 5. Determination of siderophore Capacity of Strains *Microbacterium* sp. I2, *Arthrobacter* sp. R4, *Microbacterium* sp. K1, *Microbacterium* sp. K2.

Supplementary Figure 6. Determination of ACC ability of strains *Microbacterium* sp. I2, *Arthrobacter* sp. R4, *Microbacterium* sp. K2, *Paenarthrobacter* sp. T8 and *Microbacterium* sp. T19.

Supplementary Figure 7. The ability of five positive bacterial strains to produce ACC deaminase.

Supplementary Figure 8. The effect of PEG6000 with different concentrations on the growth of strains *Microbacterium* sp. I2, *Arthrobacter* sp. R4, *Microbacterium* sp. K2.

Note: Different lowercase letters indicate significant differences in the OD600 values of the tested strains at different concentrations of PEG6000 (P<0.05)

Supplementary Figure 1


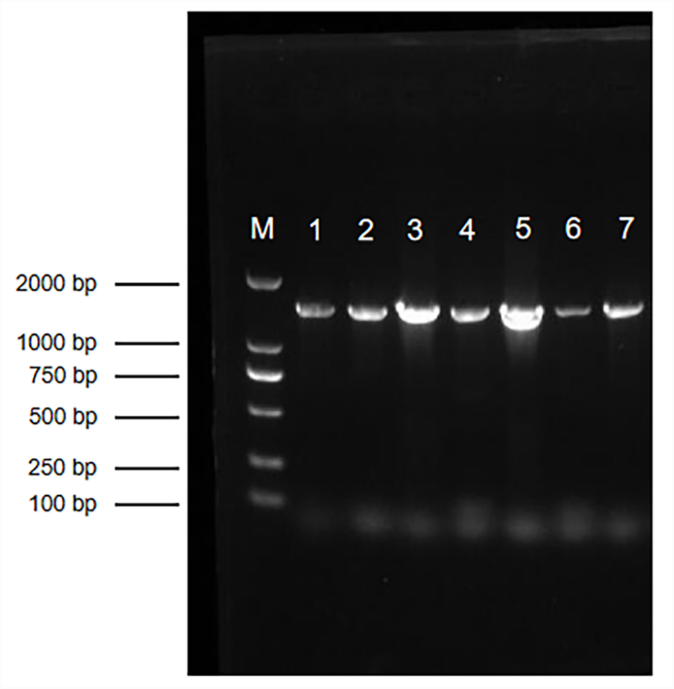


Supplementary Figure 2


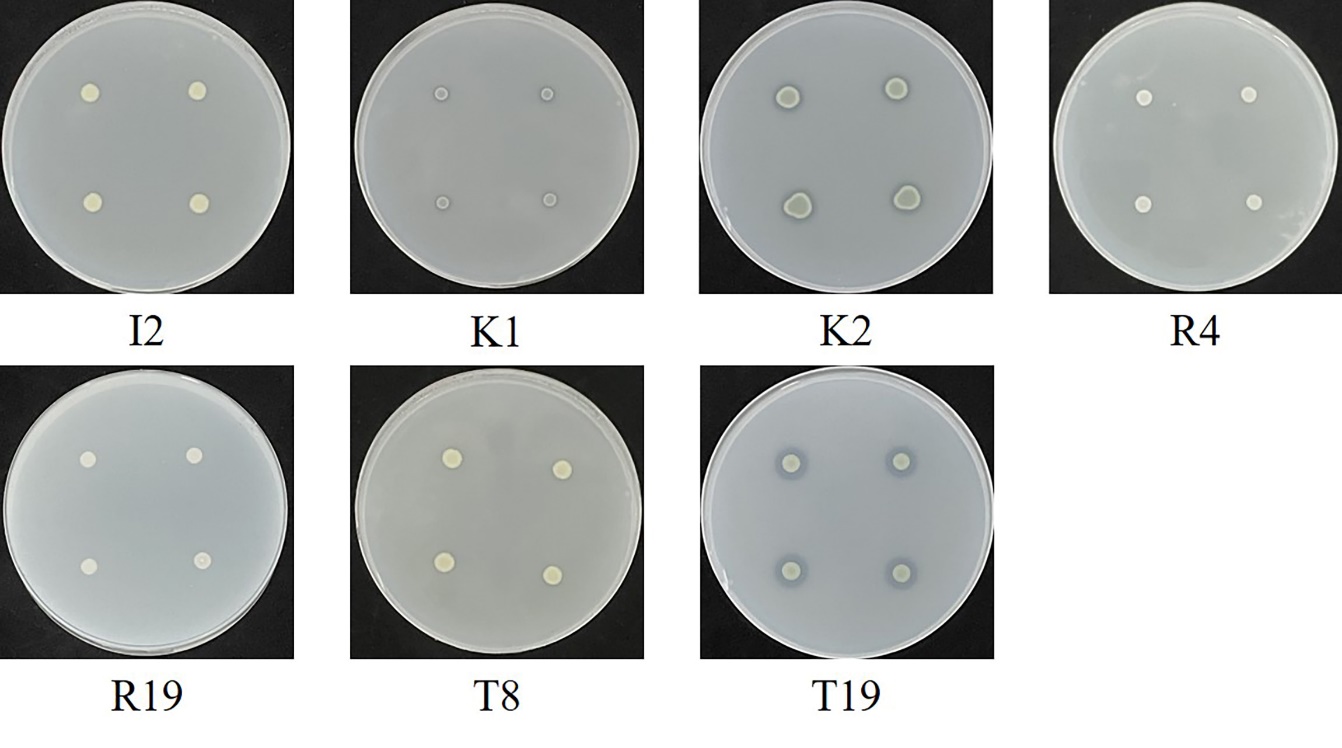


Supplementary Figure 3


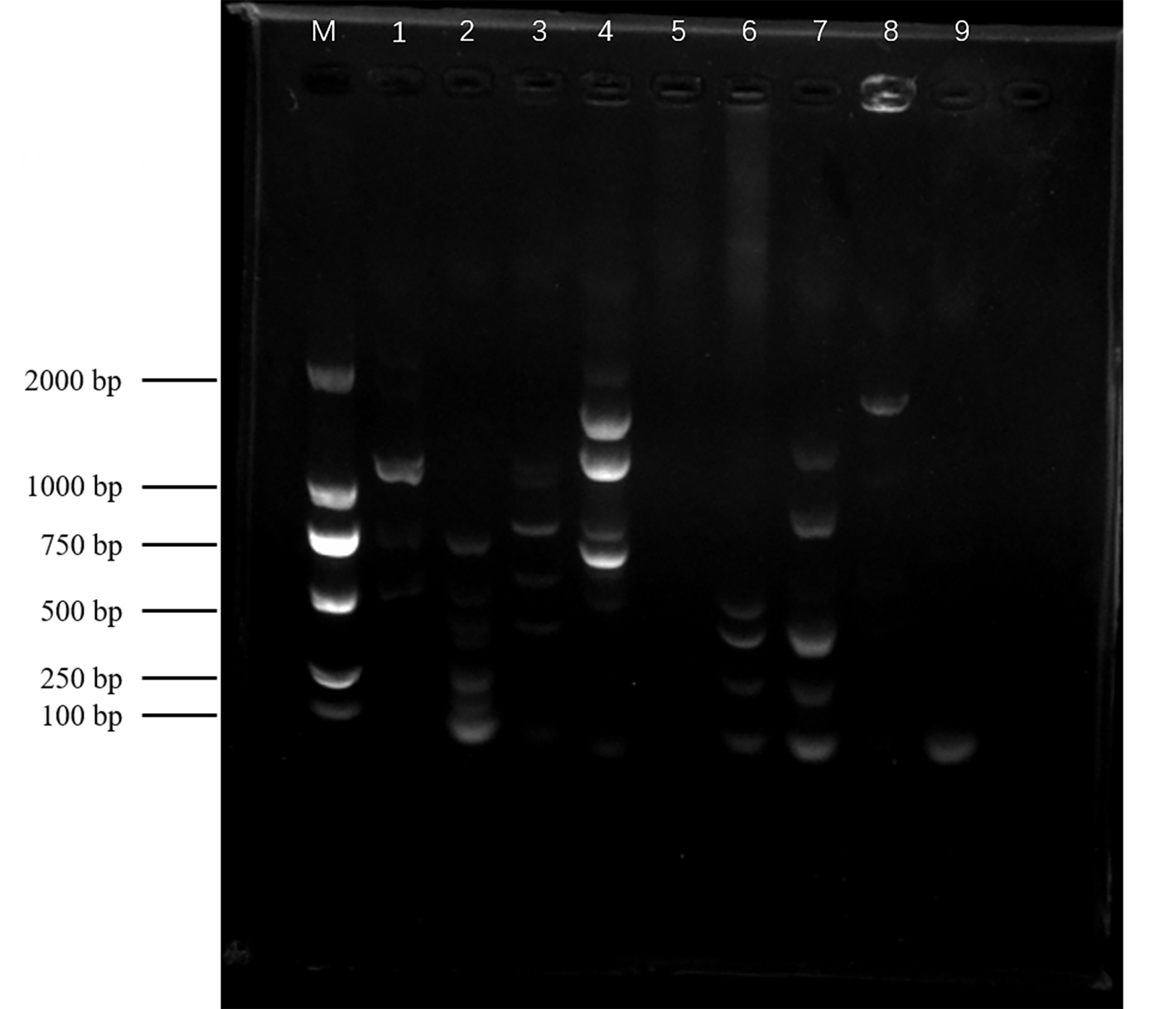


Supplementary Figure 4


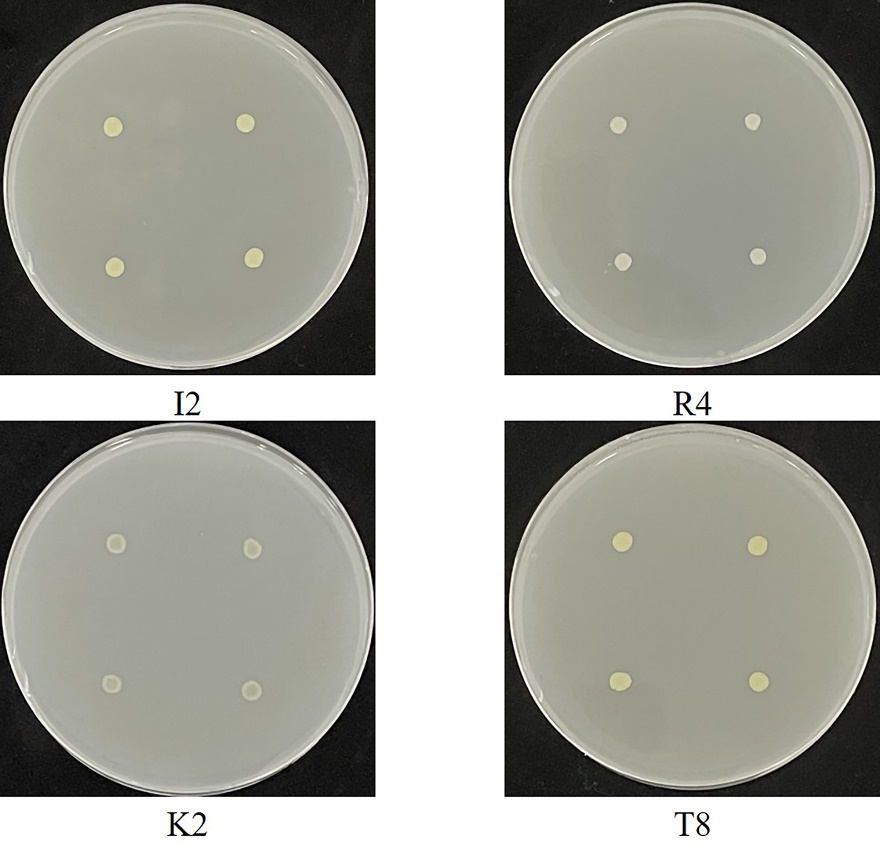


Supplementary Figure 5


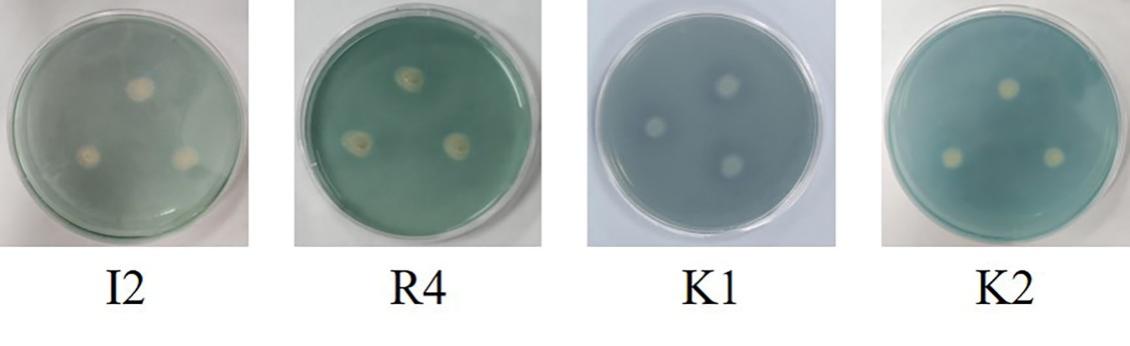


Supplementary Figure 6


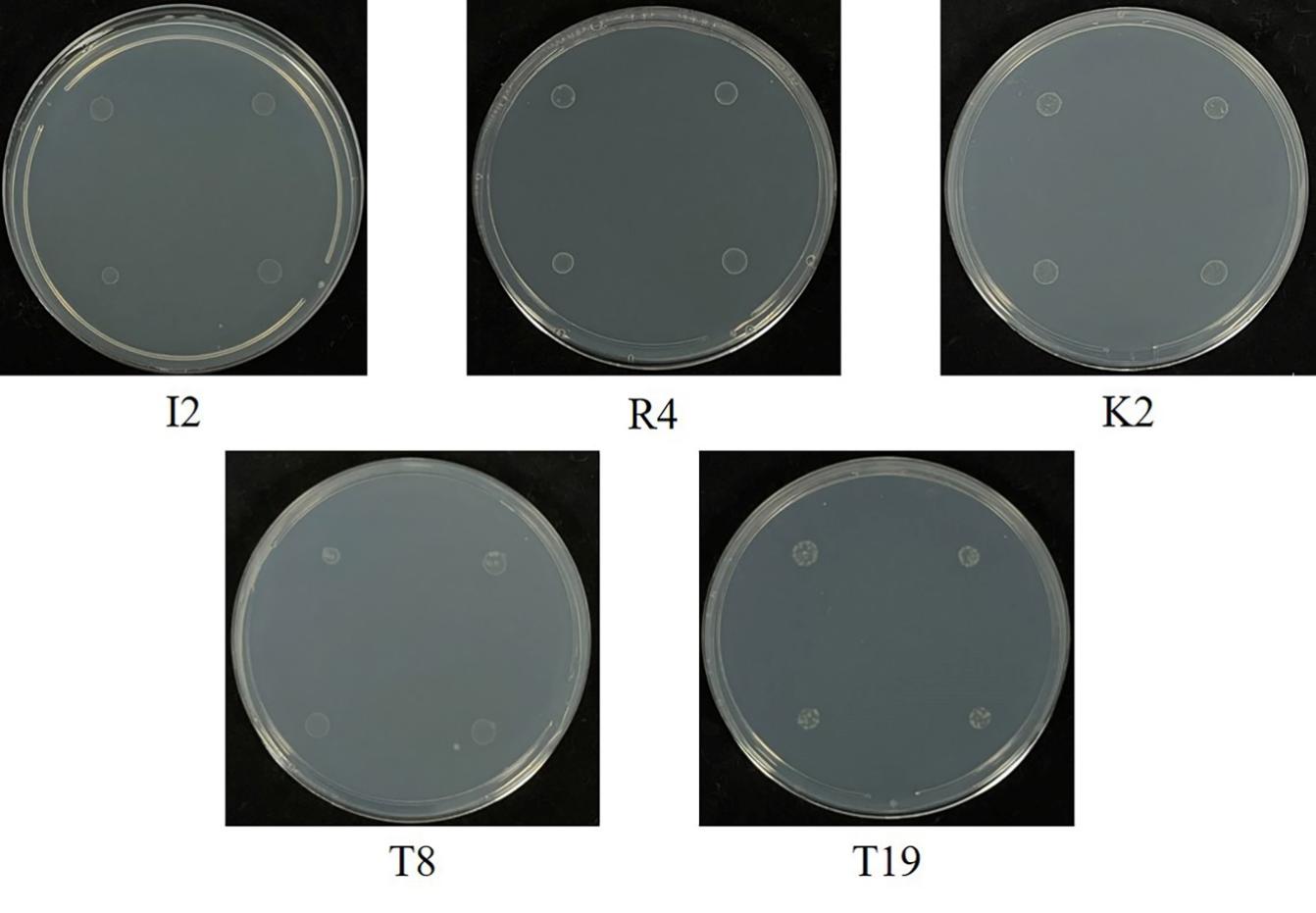


Supplementary Figure 7


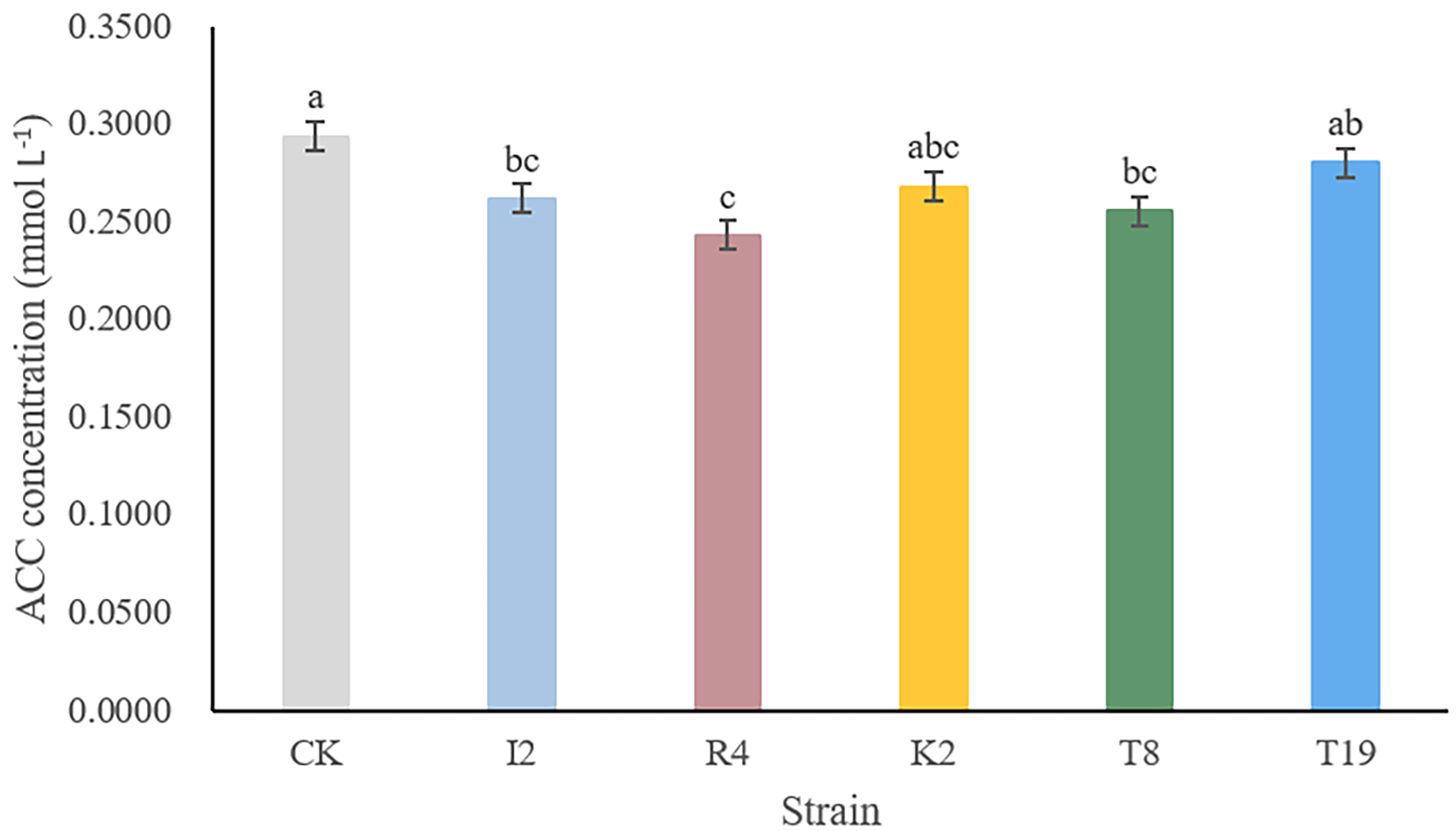


Supplementary Figure 8
