# Supplementary material for: Study on the isolation of rhizosphere bacteria and the mechanism of growth promotion in winter wheat in response to drought stress
Source: Front Plant Sci. 2025 Aug 19;16:1595554. doi: 10.3389/fpls.2025.1595554 (PMC12406708; doi:10.3389/fpls.2025.1595554)
Supplement: Supplementary file 2 [file Table1.docx]

Supplementary Table 1 Genome characteristics of strains *Microbacterium* sp. I2, *Arthrobacter* sp. R4 and *Microbacterium* sp. K2

|  | *Microbacterium* sp. I2 | *Arthrobacter* sp. R4 | *Microbacterium* sp. K2 |
| --- | --- | --- | --- |
| Gene total length(bp) | 3957576 | 4299747 | 3436560 |
| CDS | 3894 | 4467 | 3540 |
| tRNA | 47 | 51 | 44 |
| sRNA | 9 | 23 | 9 |
| rRNA | 2 | 5 | 4 |
| Plasmid | 0 | 5 | 0 |
